# Supplementary material for: TimeTalk uses single-cell RNA-seq datasets to decipher cell-cell communication during early embryo development
Source: Commun Biol. 2023 Sep 2;6:901. doi: 10.1038/s42003-023-05283-2 (PMC10475079; doi:10.1038/s42003-023-05283-2)
Supplement: Supplementary file 2 — Supplementary Information [file 42003_2023_5283_MOESM2_ESM.pdf]

Supplementary Note 1

In this note, we utilized two different time series models with theoretical Granger causality to generate simulation datasets for evaluating how the interpolation strategy may impact the Granger causality conclusion.

Based on the definition of Granger causality for two time series X_t and Y_t, if X_t is the Granger cause of Y_t, then

$$\sigma^2(Y_t|\overline{Y_t},\overline{X_t}) < \sigma^2(Y_t|\overline{Y_t})$$

The first time series model is two independent white noise:

$$Y_t = \epsilon_t, X_t = \eta_t, \epsilon_t \sim WN(0,1), \eta_t \sim WN(0,1) \qquad (1)$$

As X_t and Y_t are independent, we can deduce that

$$\sigma^2(Y_t|\overline{Y_t},\overline{X_t}) = \sigma^2(Y_t|\overline{Y_t}) = \sigma^2(Y_t) = 1$$

and $\sigma^2(X_t|\overline{X_t},\overline{Y_t}) = \sigma^2(X_t|\overline{X_t}) = \sigma^2(X_t) = 1$, therefore, X_t is not the Granger cause of Y_t and Y_t is not the Granger cause of X_t.

According to model (1), we can generate the simulation time series datasets with length N (we choose N=200). We sample each time series with length M (we choose M=50). Based on our previous investigation, winsz is the most influential parameter in interpolation. Therefore we varied winsz and tested how winsz influenced the imputed time series curve by interpolation strategy. We found that for model (1), the imputed datasets generated by small winsz can preserve the conclusion that neither X_t or Y_t is causally related to the other (Supplementary Fig. 20a-c). However, larger winsz can lead to false conclusions (Supplementary Fig. 20d-f). We conducted ten simulation rounds to prevent arbitrariness, each with 1000 replications of the above procedure. The results showed that a high ratio of false Granger causality still holds for larger winsz (Supplementary Fig. 20g-l, p-value were calculated by left Wilcox test).

The second time series model is two independent white noise:

$$Y_t = X_{t-1} + \epsilon_t, X_t = \eta_t + 0.5\eta_{t-1}, \epsilon_t \sim WN(0,1), \eta_t \sim WN(0,1) \qquad (2)$$

We can calculate that (the details can be found at https://www.math.pku.edu.cn/teachers/lidf/course/fts/ftsnotes/html/_ftsnotes/causal.html)

$$\sigma^2(Y_t|\overline{Y_t},\overline{X_t}) = 1,\ \sigma^2(Y_t|\overline{Y_t}) \approx\ 2.1328$$

and $\sigma^2(X_t|\overline{X_t},\overline{Y_t}) = \sigma^2(X_t|\overline{X_t}) = 1$,

therefore, X_t is the Granger cause of Y_t and Y_t is not the Granger cause of X_t.

According to model (2), we can generate the simulation time series datasets with length N (we choose N=200). We replicated the procedure described in (b). We sample each time series with length M (we choose M=50). Contrary to model (1), for model (2), the imputed datasets generated by small winsz cannot preserve the conclusion that X_t is the Granger cause of Y_t and Y_t is not the Granger cause of X_t. (Supplementary Fig. 21a-c). For larger winsz, the imputed datasets can successfully preserve that X_t is the Granger cause of Y_t, but not vice versa (Supplementary Fig. 21d-f). To prevent arbitrariness, we also conducted ten simulation rounds, each involving 1000 replications of the above procedure. The high ratio of false Granger causality between Y_t and X_t still holds for larger winsz (Supplementary Fig. 21g-l).

Supplementary Figure legends

Supplementary Fig. 1 The gene expression profiles of mouse early embryo development. **a** The boxplot of mean gene expression values from integrated single-cell gene expression data. Each point represents a gene. **b** The boxplot of log2(RPKM+1) value of RNA-seq data from low-input RNA-seq data downloaded from GSE66582. **c** The boxplot of quantile normalized mean gene expression values from integrated single-cell gene expression data. **d** The boxplot of quantile normalized log2(RPKM+1) value of RNA-seq data from low-input RNA-seq data downloaded from GSE66582. Each box plot contains several elements that are described as follows: the center line represents the median; the box limits, indicated by Q1 and Q3, represent the 1st and 3rd quartile values. The upper whisker of a boxplot goes from the hinge to the largest value that is no more than 1.5 times the interquartile range (IQR) away from the hinge. The IQR is the distance between the first and third quartiles. The lower whisker goes from the hinge to the smallest value which is at most 1.5 times the IQR away from the hinge. **e** Principal Component Analysis (PCA) of all collected pre-implantation scRNA-seq data and RNA-seq data.

Supplementary Fig. 2 The scatter plot of bulk and pseudo-bulk gene expression. In each subfigure, dots represent individual genes, and the x-axis represents pseudobulk gene expression, while the y-axis represents bulk gene expression. The arrangement of panels in each subfigure corresponds to different development stages. Pearson correlation coefficient (R) was used for analysis.

Supplementary Fig. 3 The kBET analysis of merged early embryo scRNA-seq data. **a** The boxplot of observed and expected rejection rates for merged early embryo scRNA-seq data. **b** The summary of the test results (with the 95% confidence interval). Q1 represents the lower quartile, Q2 represents the median, and Q3 represents the upper quartile.

Supplementary Fig. 4 The global expression dynamics of ligand and receptor genes. a The means of gene expression during early embryo development. **b** The standard derivation of gene expression during early embryo development. **c** The coefficients of variation of gene expression during early embryo development. For each boxplot, the upper half features a grey box for all genes, with a yellow box inside for the ligand gene. The lower half has a grey box for all genes, with a green box for the receptor gene. Each gene metric is denoted by a point on the bar with a mean value. The upper and lower box limits represent 1st and 3rd quartile values. The upper whisker of a boxplot goes from the hinge to the largest value that is no more than 1.5 times the interquartile range (IQR) away from the hinge. The IQR is the distance between the first and third quartiles. The lower whisker goes from the hinge to the smallest value which is at most 1.5 times the IQR away from the hinge.

Supplementary Fig. 5 The dynamics of ligand and receptor gene expression in certain eLRs reported previously. a-e The co-varying dynamics of *Fgf4-Fgfr1*, *Bmp6-Bmpr1b*, *Adam17-Cd9*, *Pdgfc-Pdgfra*, *Igf2-Igf2r*. PCC: Pearson's correlation coefficient, SCC: Spearman's correlation coefficient.

Supplementary Fig. 6 The dynamics of ligand-receptor activity and tTFs. The dynamics of the activity of *Fgf4-Fgfr1* and tTFs *Gata6* (**a**), *Pou5f1* (**b**), *Sox2* (**c**), and *Nanog* (**d**), separately. The dynamics of the activity of *Fgf4-Fgfr2* and tTFs *Gata6* (**e**), *Pou5f1* (**f**), *Sox2* (**g**), and *Nanog* (**h**), separately.

Supplementary Fig. 7 Using the tangent line strategy to determine the cutoff of co-varying ligand-receptor. a The scatterplot of sorted positive correlated LR. **b** The scatter of sorted negative correlated LR. a The scatterplot of sorted positive correlated LR. b The scatter of sorted negative correlated LR. We first rank the positively correlated LR in ascending order by PCC, producing a concave curve. The start and endpoints of this curve are labeled as A and B, respectively. We then draw a straight line connecting AB, which is moved to obtain the tangency point C. Next, we connect AC and move the

resulting straight line to obtain tangency point D. Below point D, and the curve roughly changes into a linear function. Thus, we select the vertical coordinate of point D as the cutoff value, which is 0.0987, for positive co-varying LR (**a**). Using the same process, the cutoff for the negative co-varying LR is -0.0587 (**b**).

Supplementary Fig. 8 The dynamics of the different clusters of tTFs. a-e The dynamics of the cluster of tTFs C1, C2, C3, C4, and C6. These figures are related to Fig. 2f and Fig. 2h.

Supplementary Fig. 9 The dynamics of *Bmp4-Bmpr2* activity and the activity of different clusters of tTFs. **a-f** The line plots demonstrate the dynamics of *Bmp4-Bmpr2* activity and the activity of different clusters of tTFs. Different clusters were distinguished by different colors. The activity of different clusters of tTFs was quantified by the mean value gene expression of each cluster of tTFs.

Supplementary Fig. 10 The dynamics of *Fgf4-Fgfr2* activity to tTFs and the activity in C2, C3, C4, C6 cluster. a-e The line plots demonstrate the dynamics of *Fgf4-Fgfr2* activity to tTFs and the activity in C2, C3, C4, C6 cluster. Different clusters were distinguished by different colors. The activity of different clusters of tTFs was quantified by the mean value gene expression of each cluster of tTFs.

Supplementary Fig. 11 The overlap of different gene sets with eLR. a The Venn diagram of essential gene overlap with LR and eLR. **b** The Venn diagram of the Housekeeping gene overlaps with LR, eLR. **c** The Venn diagram of the ZGA gene overlaps with LR and eLR. The grey box represents the protein-coding gene set composed of 24230 protein-coding genes.

Supplementary Fig. 12 The identification of eLR affected by ZGA inhibition treatment. Two rounds of hierarchical clustering identified the eLR pairs affected by ZGA inhibition. Euclidean distance and complete clustering methods were used for

hierarchical clustering. In the first step, the variable eLR pairs were identified. The second step involved identifying the eLR pairs that were affected by ZGA inhibition. The eLR pairs identified in each step are highlighted in a red dashed box. The unit used for the color gradient keys in Supplementary Fig 12 is log10(IS+1).

Supplementary Fig. 13 The comparison of the impact of ZGA inhibition treatment on eLRs and non-eLRs. The delta values of ZGA inhibition on eLR and non-eLRs are displayed in a violin plot. Each dot on the plot represents a ligand-receptor pair, with eLR and non-eLR pairs being distinguished by different colors. The black point in each plot represents the mean value of each group, which has been highlighted. The t-test was used to calculate the p-value. To determine whether the delta value of non-eLR was lower than that of eLR, a left-tailed t-test was employed. For each group, the error bar interval was calculated as [mean-sd, mean+sd]. The "mean" refers to the average value of the delta value for each LR pair in the group, while "sd" represents the standard error of the delta value for each LR pair in that same group.

Supplementary Fig. 14 The maternal factor and ZGA gene overlap ratio. a The ratio of maternal factors contained in genes composed of the different clusters of eLR pairs. **b** The ratio of the ZGA gene contained in genes composed of the different clusters of eLR pairs.

Supplementary Fig. 15 Further investigation of eLRs enriched in the Hippo signaling pathway within clusters 4 and 6 a The list of eLR enriched in the Hippo signaling pathway within clusters 4 and clusters 6. **b** The dynamics of *Wnt3a-Fzd5* and *Tead2.* **c** The dynamics of *Cthrc1-Fzd3* and *Tead4*.

Supplementary Fig. 16 The ratio of the different clusters of eLR regulates tTFs. The grouped bar charts depict the proportion of eLRs in each eLR class that can potentially regulate tTFs. The grouped bar chart illustrates the results obtained from TimeTalk (referred to as "infer") and the prior relationships collected in the NicheNet database (referred to as "NicheNet"). These figures are related to Fig. 4c, Fig. 4d, and Fig. 4e.

Supplementary Fig. 17 The percent of the different clusters of tTFs regulate eLR. The bar charts demonstrate, in different categories of tTFs, the ratio of tTFs that regulate a large number of eLRs and tTFs that regulate a small number of eLRs. The criterion for a large number is that the proportion of eLR genes in the corresponding target genes of each tTF exceeds 5%, which is considered as tTFs with a large number of eLR targets. This figure is related to Fig. 4f.

Supplementary Fig. 18 The re-analysis of the blastocyst and blastoid scRNA-seq datasets. a The t-SNE embedding of the integrated blastocyst and blastoid data. **b** The UMAP embedding of the integrated blastocyst and blastoid data. **c** The feature plot of marker expression of different lineage. **d** The feature plot of 2-cell genes *Zscan4c*, *Zscan4d*, and *Zscan4f*. **e** The feature plot 2C score. The unit for color gradient keys in **c-e** is LogNormalize, which involves dividing the feature counts for each cell by the total counts for that cell and then multiplying it by the scale.factor. This value is then transformed using the natural-log function and adding 1. **f** The cell type annotation of integrated blastoid and blastocyst datasets. **g** The statistics of cell type composition in blastocyst and blastoids.

Supplementary Fig. 19 The sensitivity and reliability analysis of TimeTalk. a The barplot displays the overlapped ratio while keeping numPts and lags fixed but varying winsz.**b** The barplot displays the overlapped ratio while keeping winsz and lags fixed but varying lags. **c** The barplot displays the overlapped ratio while keeping winsz and Numpts fixed but varying lags. **d**. The heatmap displays the overlapped ratio for different combinations of winsz, numPts, and lags. **e** The Interpolated dynamic Curves for *Fgf4-Fgfr2* and *Gata6*. **f** The Interpolated dynamic Curves for *Fgf4* and *Nanog.* **g** The interpolated dynamic curves of *Fgfr2* and *Gata6.*

Supplementary Fig. 20 Evaluating the performance of interpolation and Granger causal inference on simulation data without Granger causality. a-f The line plot of

time series and interpolated time series, while fixing the other two parameters and varying the winsz. **g-h** The ratio of false positives of 10 rounds simulation results with different winsz. The details are described in Supplementary Note 1.

Supplementary Fig. 21 Evaluating the performance of interpolation and Granger causal inference on simulation data with Granger causality. a-f The line plot of time series and interpolated time series, while fixing the other two parameters and varying the winsz. **g-h** The ratio of false positives of 10 rounds simulation results with different winsz. The details are described in Supplementary Note 1.

Supplementary Fig. 22. **The co-evolution analysis of LR and non-eLR. a** The boxplot of correlation of the Ka/Ks ratio of ligand gene and receptor gene in the eLR and non-eLR groups. The p-value was calculated by two-sided Wilcoxon test. **b** The correlation of Ka/Ks ratio of eLR *Fgf10-Fgfr2*. **c** The correlation of Ka/KS ratio of non-eLR *Ccl21b-Cxcr3*.

Supplementary Fig. 1: The gene expression profiles of mouse early embryo development

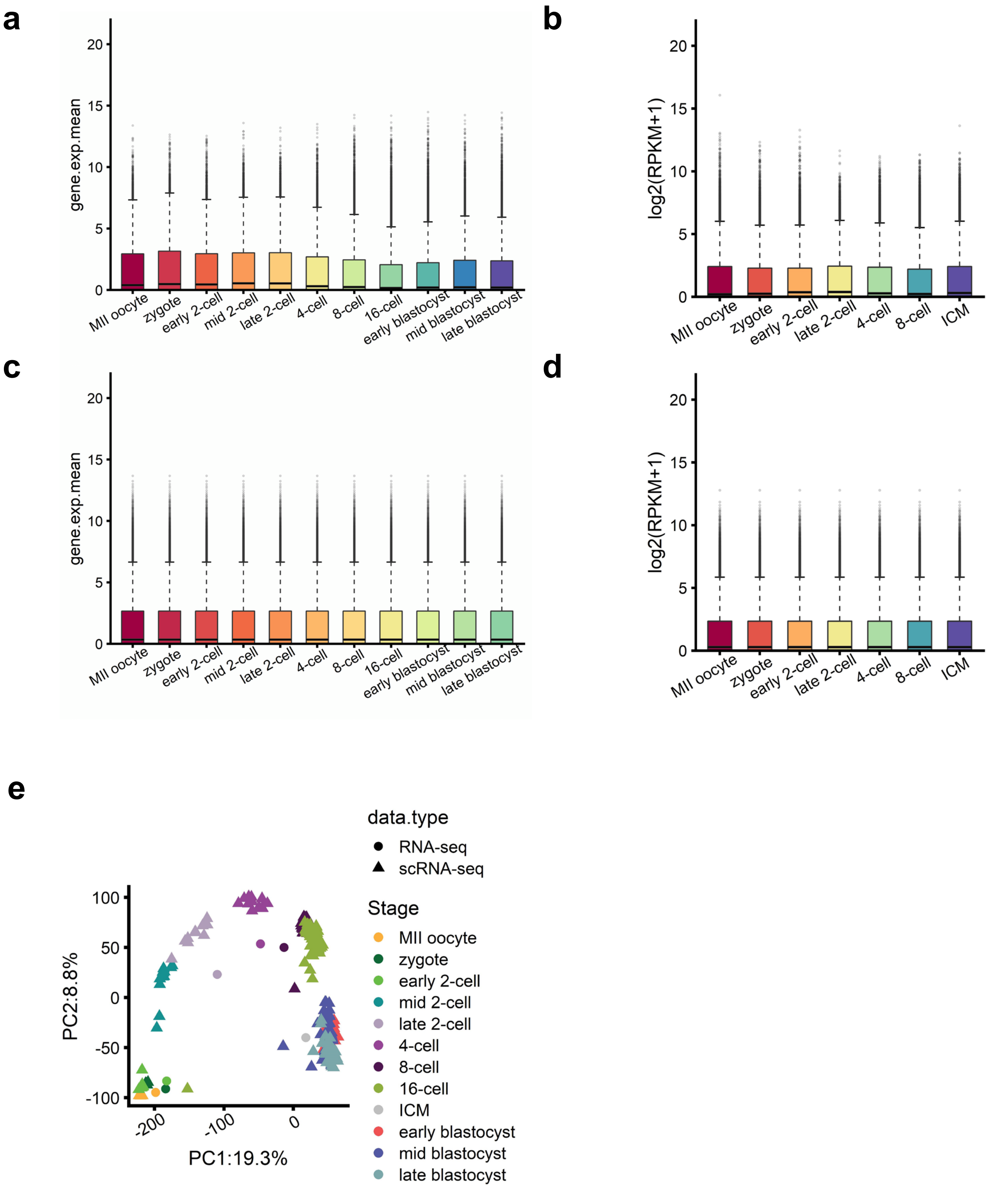

a The boxplot of mean gene expression values from integrated single-cell gene expression data. Each point represents a gene. **b** The boxplot of log2(RPKM+1) value of RNA-seq data from low-input RNA-seq data downloaded from GSE66582. **c** The boxplot of quantile normalized mean gene expression values from integrated single-cell gene expression data. **d** The boxplot of quantile normalized log2(RPKM+1) value of RNA-seq data from low-input RNA-seq data downloaded from GSE66582. Each box plot contains several elements that are described as follows: the center line represents the median; the box limits, indicated by Q1 and Q3, represent the 1st and 3rd quartile values. The upper whisker of a boxplot goes from the hinge to the largest value that is no more than 1.5 times the interquartile range (IQR) away from the hinge. The IQR is the distance between the first and third quartiles. The lower whisker goes from the hinge to the smallest value which is at most 1.5 times the IQR away from the hinge. **e** Principal Component Analysis (PCA) of all collected pre-implantation scRNA-seq data and RNA-seq data.

Supplementary Fig. 2: The scatter plot of bulk and pseudo-bulk gene expression

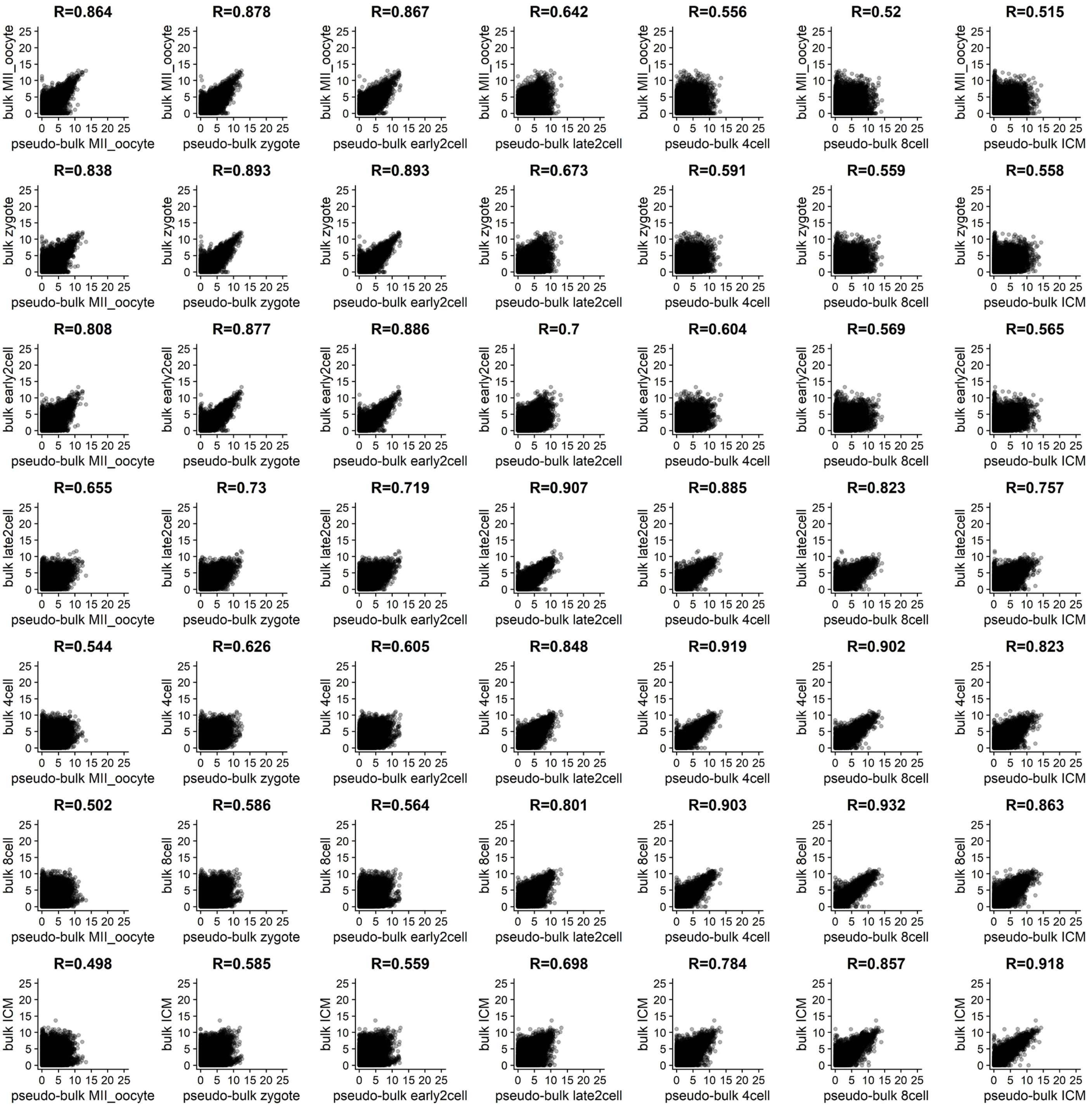

In each subfigure, dots represent individual genes, and the x-axis represents pseudobulk gene expression, while the y-axis represents bulk gene expression. The arrangement of panels in each subfigure corresponds to different development stages. Pearson correlation coefficient (R) was used for analysis.

Supplementary Fig. 3: The kBET analysis of merged early embryo scRNA-seq data

a

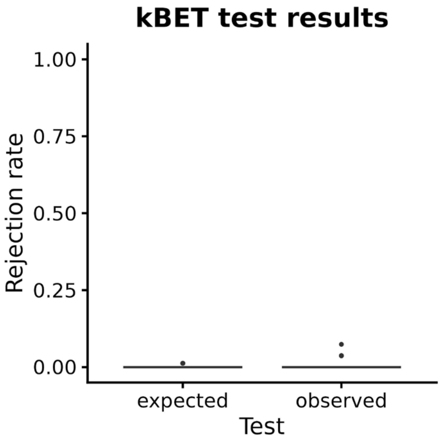

b

kBET_quantile	kBET.expected	kBET.observed	kBET.signif
mean	0.001111111	0.009259259	0.8611531
Q1	0	0	0.3074577
Q2	0	0	1
Q3	0.012345679	0.037037037	1

a The boxplot of observed and expected rejection rates for merged early embryo scRNA-seq data. **b** The summary of the test results (with the 95% confidence interval). Q1 represents the lower quartile, Q2 represents the median, and Q3 represents the upper quartile.

Supplementary Fig. 4: The global expression dynamics of ligand and receptor genes

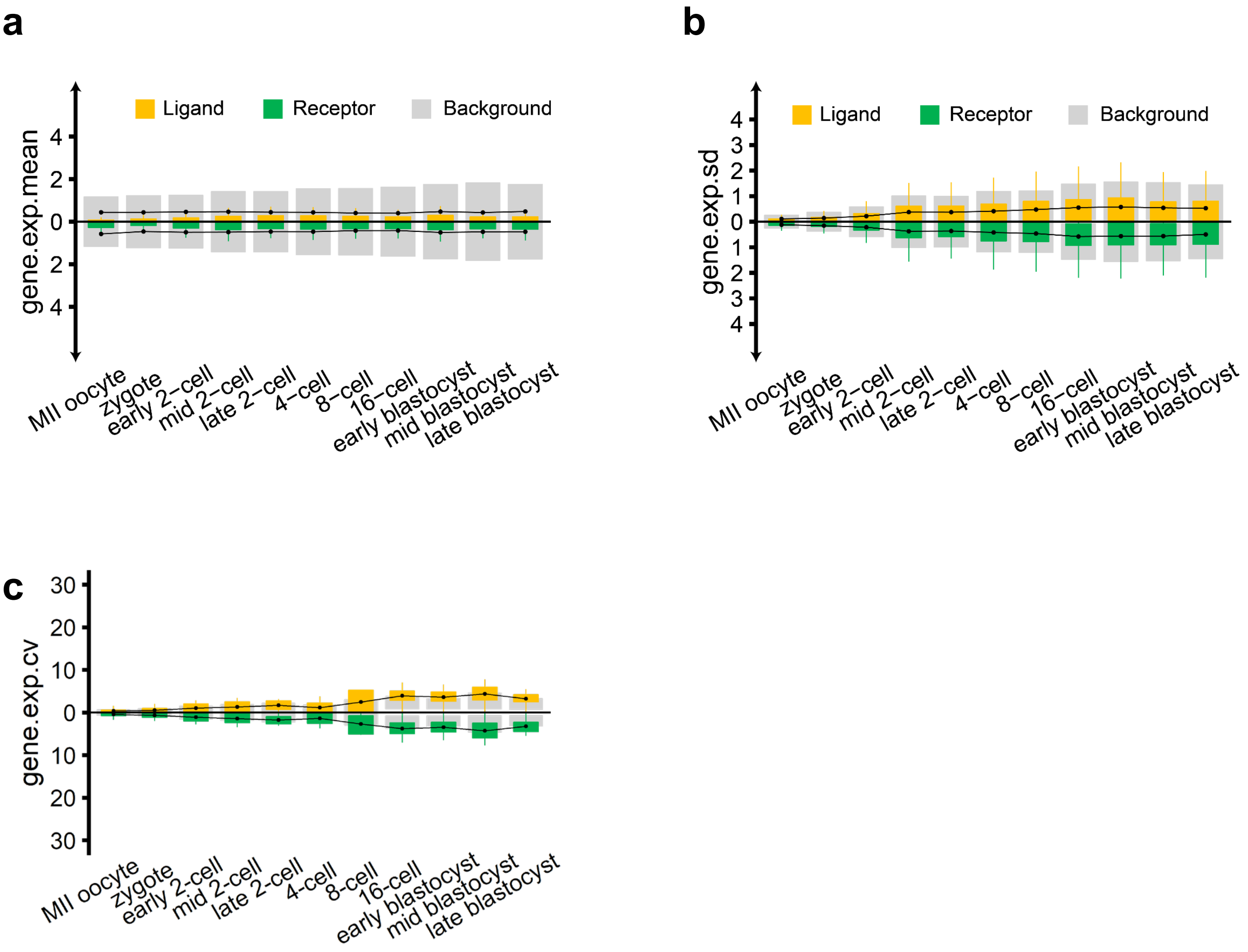

a The means of gene expression during early embryo development. **b** The standard derivation of gene expression during early embryo development. **c** The coefficients of variation of gene expression during early embryo development. For each boxplot, the upper half features a grey box for all genes, with a yellow box inside for the ligand gene. The lower half has a grey box for all genes, with a green box for the receptor gene. Each gene metric is denoted by a point on the bar with a mean value. The upper and lower box limits represent 1st and 3rd quartile values. The upper whisker of a boxplot goes from the hinge to the largest value that is no more than 1.5 times the interquartile range (IQR) away from the hinge. The IQR is the distance between the first and third quartiles. The lower whisker goes from the hinge to the smallest value which is at most 1.5 times the IQR away from the hinge.

Supplementary Fig. 5: The dynamics of ligand and receptor gene expression in certain eLRs reported previously

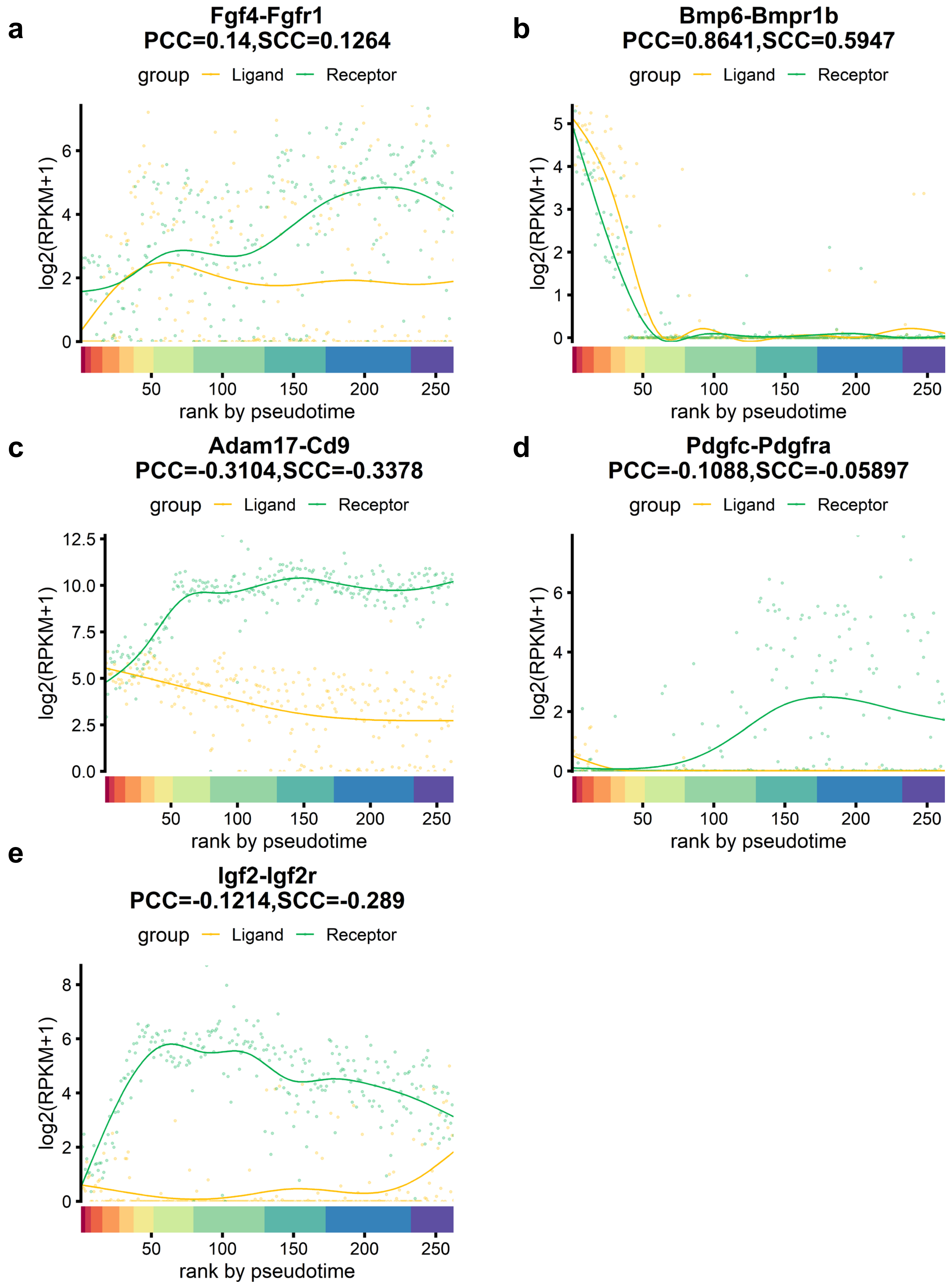

a-e The co-varying dynamics of *Fgf4-Fgfr1***,** *Bmp6-Bmpr1b*, *Adam17-Cd9*, *Pdgfc-Pdgfra*, *Igf2-Igf2r*. PCC: Pearson's correlation coefficient, SCC: Spearman's correlation coefficient.

Supplementary Fig. 6: The dynamics of ligand-receptor activity and tTFs

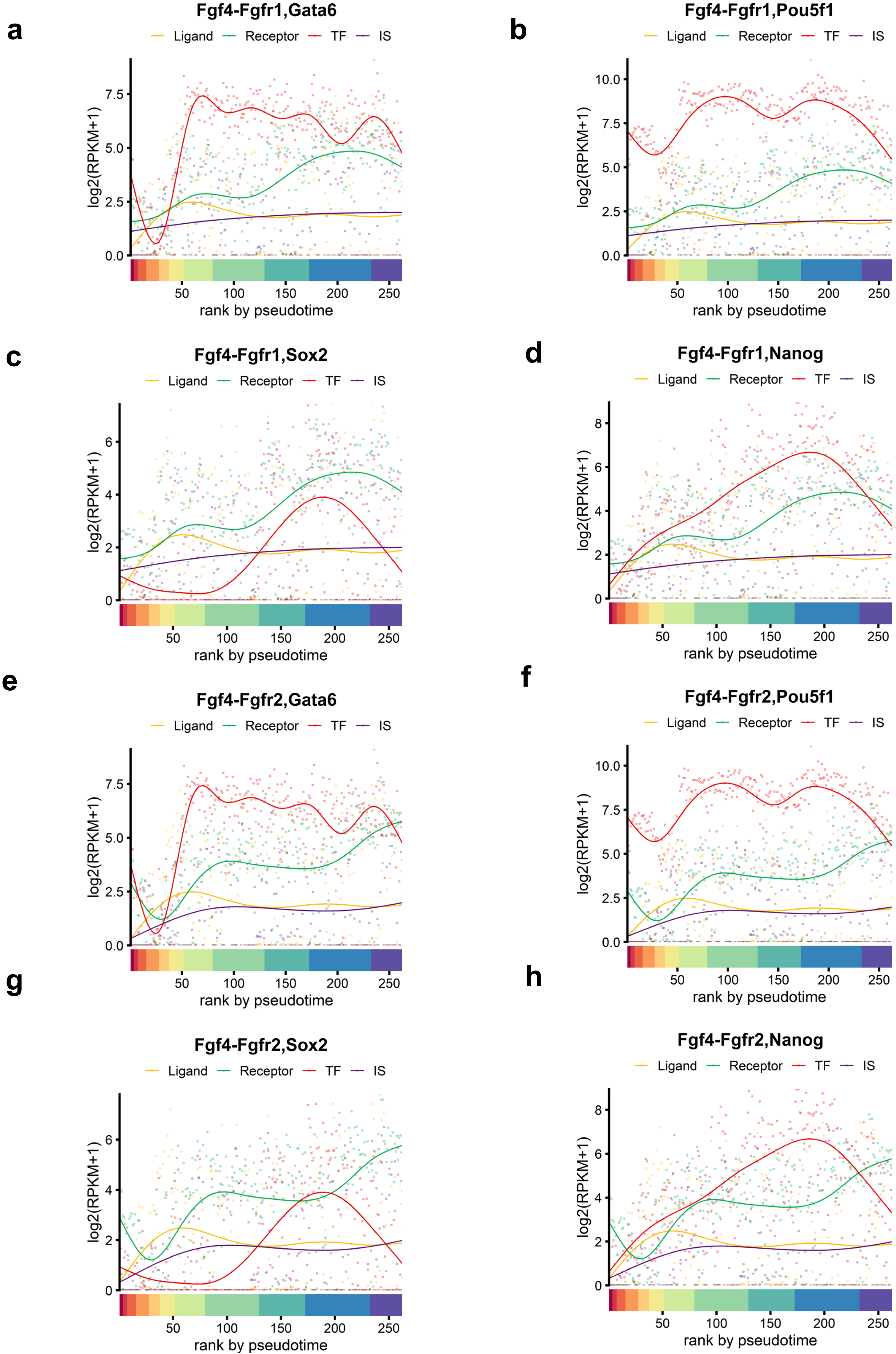

The dynamics of the activity of *Fgf4-Fgfr1* and tTFs *Gata6* (**a**), *Pou5f1* (**b**), *Sox2* (**c**), and *Nanog* (**d**), separately. The dynamics of the activity of *Fgf4-Fgfr2* and tTFs *Gata6* (**e**), *Pou5f1* (**f**), *Sox2* (**g**), and *Nanog* (**h**), separately.

Supplementary Fig. 7: Using the tangent line strategy to determine the cutoff of co-vary ligand-receptor

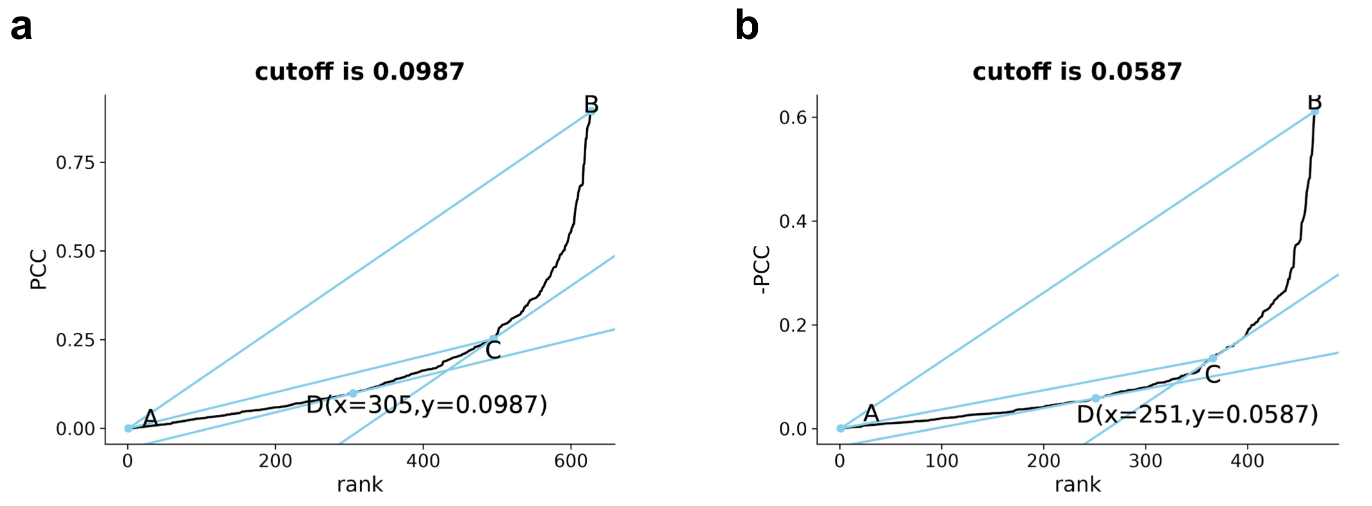

a The scatterplot of sorted positive correlated LR. **b** The scatter of sorted negative correlated LR. We first rank the positively correlated LR in ascending order by PCC, producing a concave curve. The start and endpoints of this curve are labeled as A and B, respectively. We then draw a straight line connecting AB, which is moved to obtain the tangency point C. Next, we connect AC and move the resulting straight line to obtain tangency point D. Below point D, and the curve roughly changes into a linear function. Thus, we select the vertical coordinate of point D as the cutoff value, which is 0.0987, for positive co-varying LR (**a**). Using the same process, the cutoff for the negative co-varying LR is -0.0587 (**b**).

Supplementary Fig. 8: The dynamics of the different clusters of tTFs

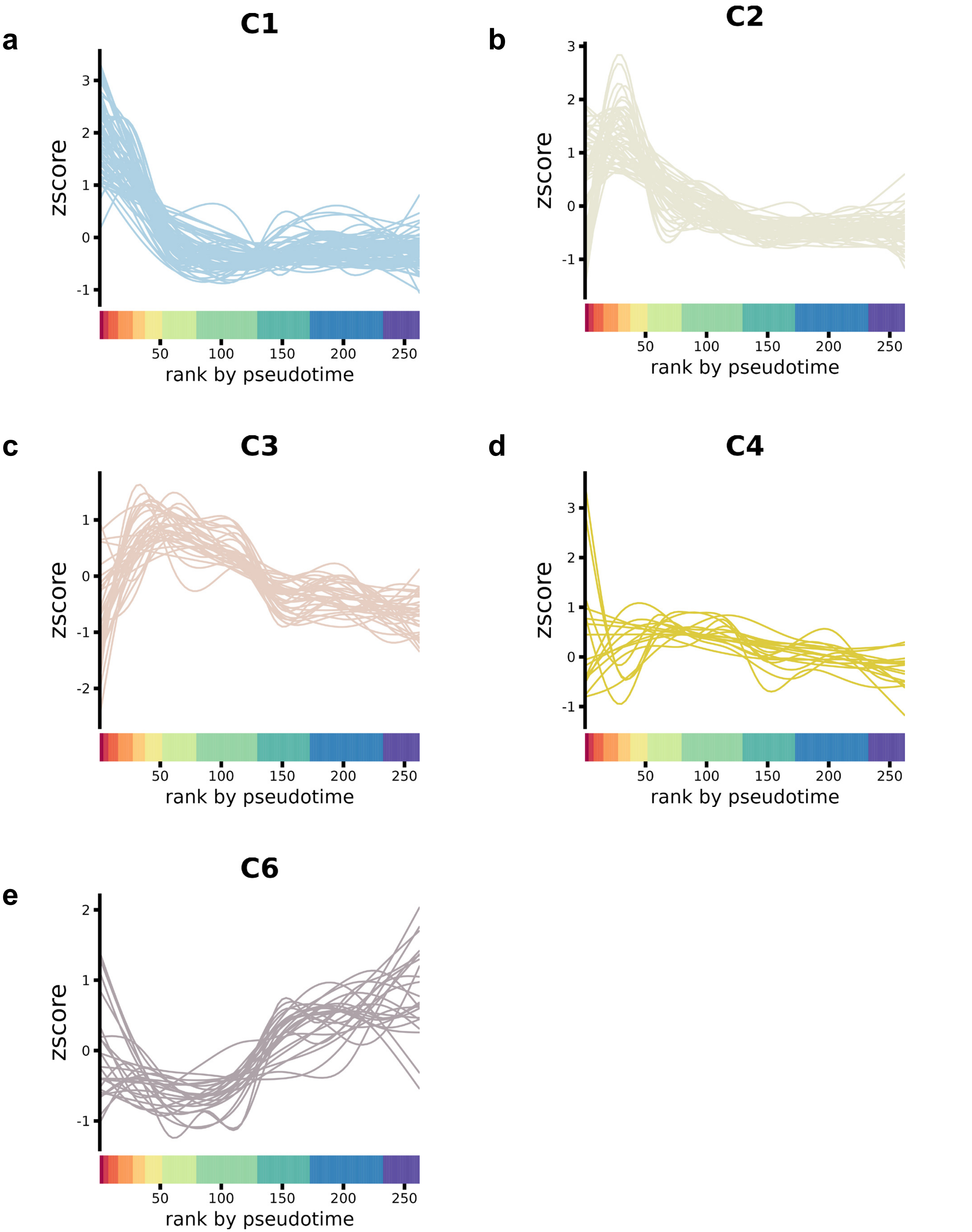

a-e The dynamics of the cluster of tTFs C1, C2, C3, C4, and C6. These figures are related to Fig. 2f and Fig. 2h.

Supplementary Fig. 9: The dynamics of *Bmp4-Bmpr2* activity and the activity of different clusters of tTFs

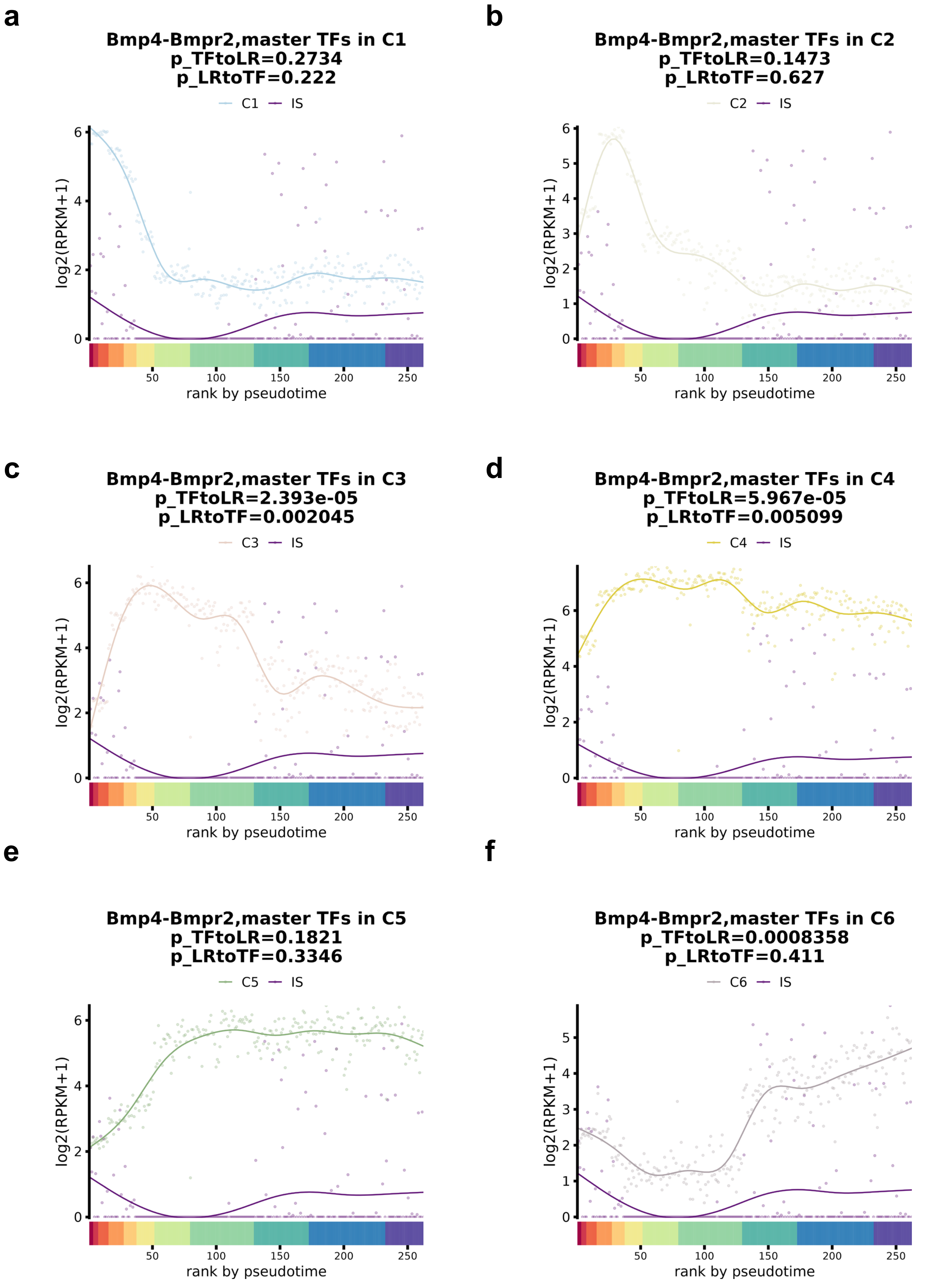

a-f The line plots demonstrate the dynamics of *Bmp4-Bmpr2* activity and the activity of different clusters of tTFs. Different clusters were distinguished by different colors. The activity of different clusters of tTFs was quantified by the mean value gene expression of each cluster of tTFs.

Supplementary Fig. 10: The dynamics of *Fgf4-Fgfr2* activity to tTFs and the activity in C2, C3, C4, C6 cluster

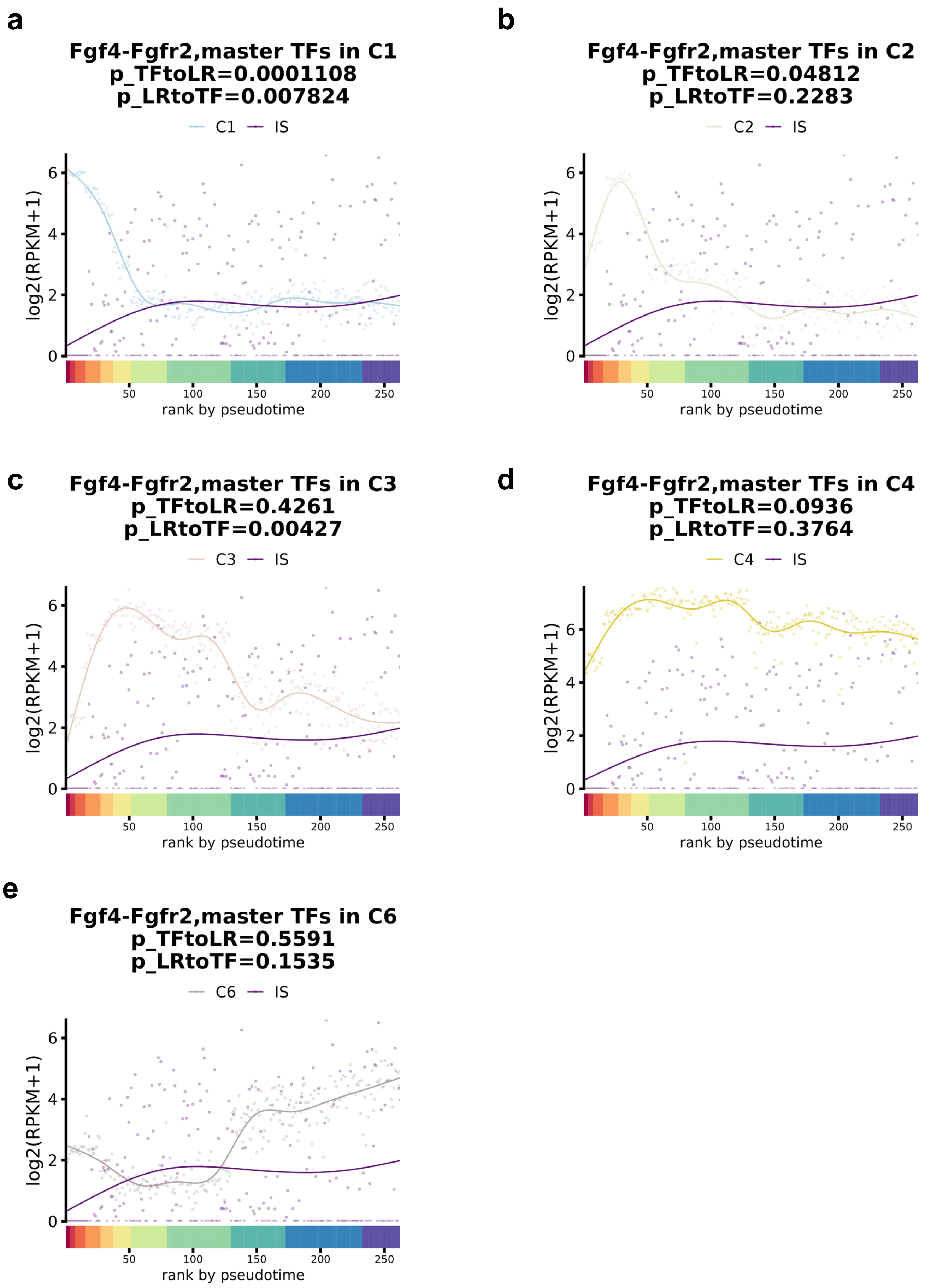

a-e The line plots demonstrate the dynamics of *Fgf4-Fgfr2* activity to tTFs and the activity in C2, C3, C4, C6 cluster. Different clusters were distinguished by different colors. The activity of different clusters of tTFs was quantified by the mean value gene expression of each cluster of tTFs.

Supplementary Fig. 11: The overlap of different gene sets with eLR

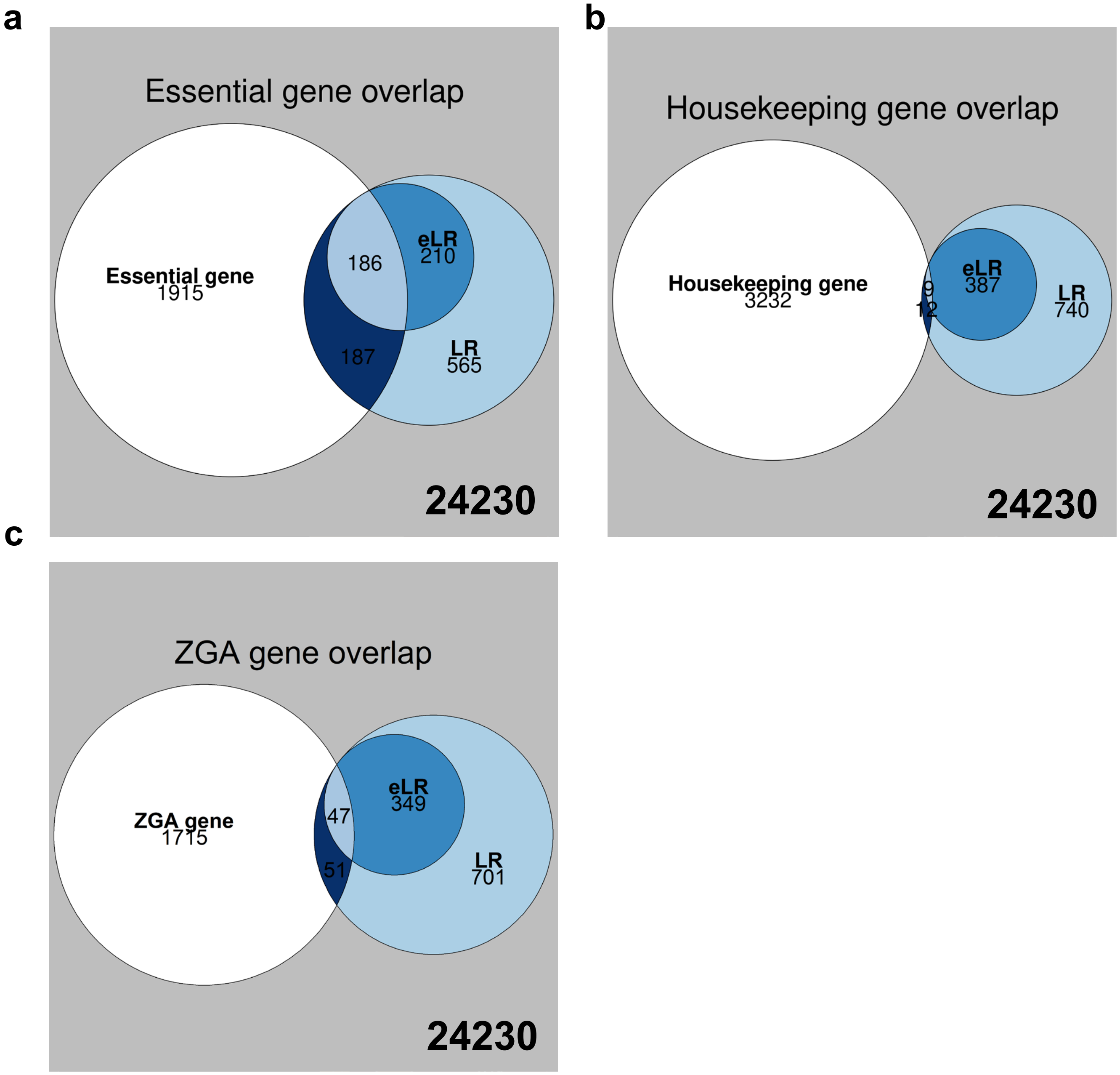

a The Venn diagram of essential gene overlap with LR and eLR. **b** The Venn diagram of the Housekeeping gene overlaps with LR, eLR. **c** The Venn diagram of the ZGA gene overlaps with LR and eLR. The grey box represents the protein-coding gene set composed of 24230 protein-coding genes.

Supplementary Fig. 12: The identification of eLRs affected by ZGA inhibition treatment

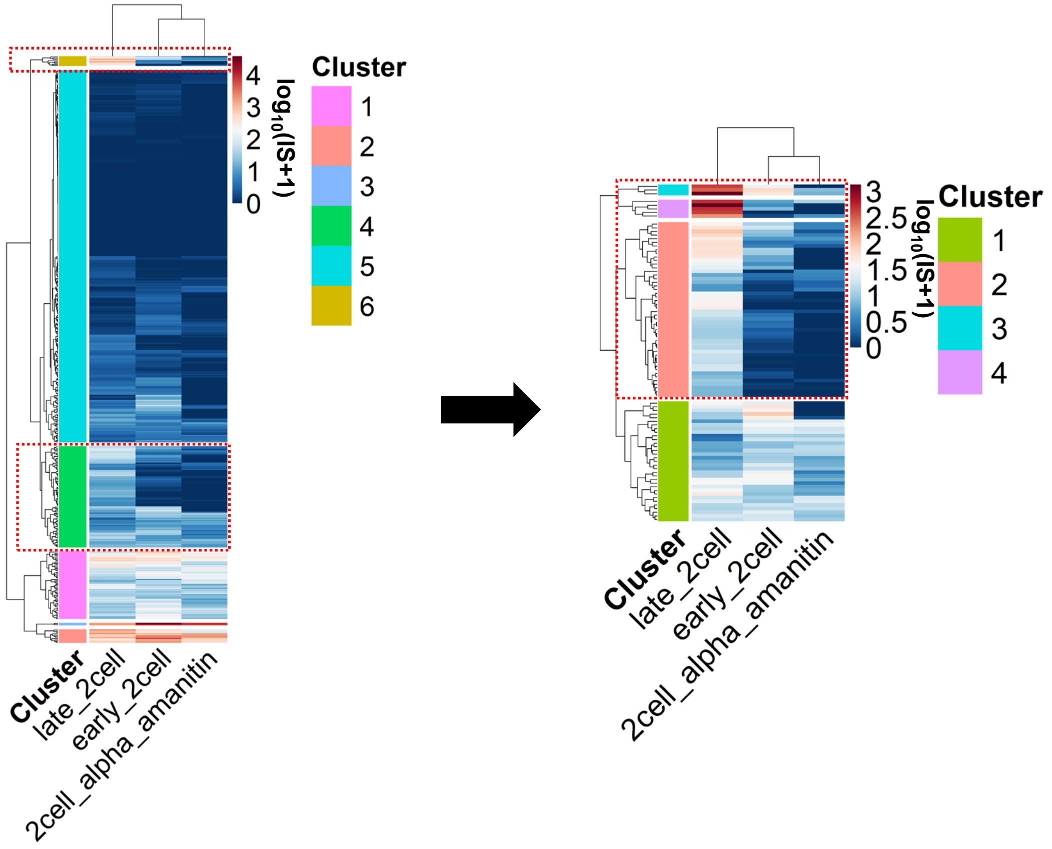

Two rounds of hierarchical clustering identified the eLR pairs affected by ZGA inhibition. Euclidean distance and complete clustering methods were used for hierarchical clustering. In the first step, the variable eLR pairs were identified. The second step involved identifying the eLR pairs that were affected by ZGA inhibition. The eLR pairs identified in each step are highlighted in a red dashed box. The unit used for the color gradient keys in Supplementary Fig 12 is log10(IS+1).

Supplementary Fig. 13: The comparison of the impact of ZGA inhibition treatment on eLRs and non-eLRs.

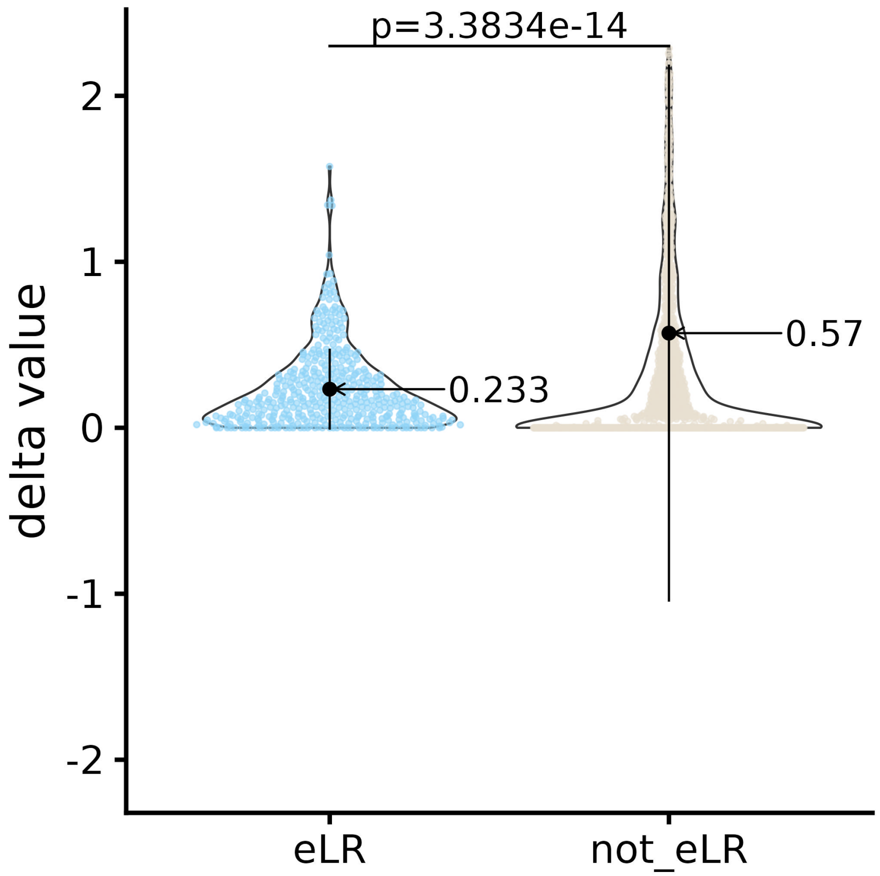

The delta values of ZGA inhibition on eLR and non-eLRs are displayed in a violin plot. Each dot on the plot represents a ligand-receptor pair, with eLR and non-eLR pairs being distinguished by different colors. The black point in each plot represents the mean value of each group, which has been highlighted. The t-test was used to calculate the p-value. To determine whether the delta value of non-eLR was lower than that of eLR, a left-tailed t-test was employed. For each group, the error bar interval was calculated as [mean-sd, mean+sd]. The "mean" refers to the average value of the delta value for each LR pair in the group, while "sd" represents the standard error of the delta value for each LR pair in that same group.

Supplementary Fig. 14: The maternal factor and ZGA gene overlap ratio

a

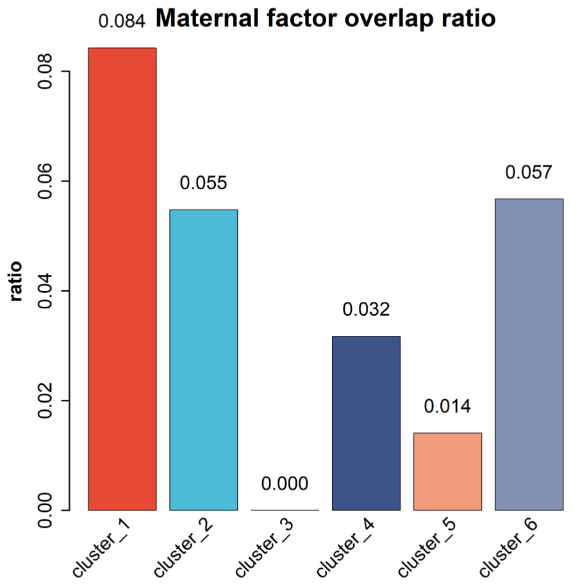

b

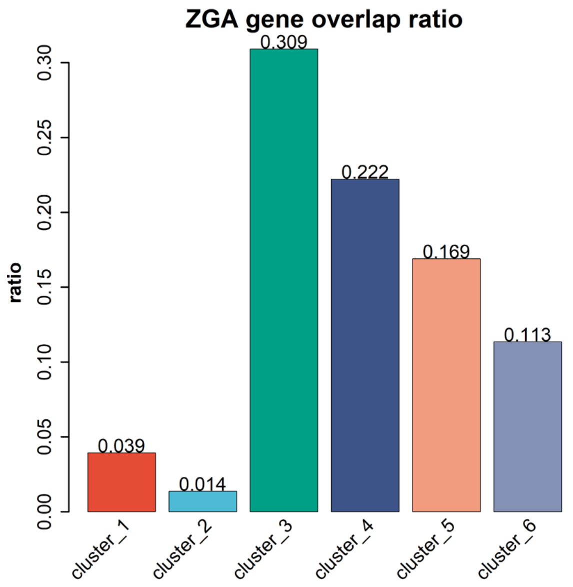

a The ratio of maternal factors contained in genes composed of the different clusters of eLR pairs. **b** The ratio of the ZGA gene contained in genes composed of the different clusters of eLR pairs.

Supplementary Fig. 15: Further investigation of eLRs enriched in the Hippo signaling pathway within clusters 4 and 6

a

eLR	L_gene	R_gene	Cluster
Cdh1-Itgb7	Cdh1	Itgb7	cluster_4
Cdh1-Itgae	Cdh1	Itgae	cluster_4
Gdf3-Tgfbr1	Gdf3	Tgfbr1	cluster_4
Tdgf1-Tgfbr1	Tdgf1	Tgfbr1	cluster_4
Wnt3a-Fzd2	Wnt3a	Fzd2	cluster_4
Icam2-Itgb2	Icam2	Itgb2	cluster_4
Tln1-Itgb2	Tln1	Itgb2	cluster_4
Plau-Itgb2	Plau	Itgb2	cluster_4
Areg-Egfr	Areg	Egfr	cluster_4
Gdf5-Ror2	Gdf5	Ror2	cluster_4
Wnt5a-Fzd5	Wnt5a	Fzd5	cluster_4
Wnt7a-Fzd5	Wnt7a	Fzd5	cluster_4
Wnt7a-Fzd1	Wnt7a	Fzd1	cluster_4
Wnt7a-Fzd4	Wnt7a	Fzd4	cluster_4
Wnt7a-Fzd9	Wnt7a	Fzd9	cluster_4
Wnt5a-Fzd4	Wnt5a	Fzd4	cluster_4
Gdf5-Acvr2b	Gdf5	Acvr2b	cluster_4
Gdf5-Bmpr1b	Gdf5	Bmpr1b	cluster_4
Gdf5-Bmpr2	Gdf5	Bmpr2	cluster_4
Fgf1-Fgfr1	Fgf1	Fgfr1	cluster_4
Fgf1-Fgfr2	Fgf1	Fgfr2	cluster_4
Cthrc1-Fzd3	Cthrc1	Fzd3	cluster_4
Cthrc1-Fzd5	Cthrc1	Fzd5	cluster_4
Wnt3a-Fzd7	Wnt3a	Fzd7	cluster_6
Wnt3a-Fzd5	Wnt3a	Fzd5	cluster_6
Wnt3a-Lrp6	Wnt3a	Lrp6	cluster_6
Wnt3a-Fzd3	Wnt3a	Fzd3	cluster_6
Wnt7b-Fzd3	Wnt7b	Fzd3	cluster_6
Wnt3a-Fzd6	Wnt3a	Fzd6	cluster_6

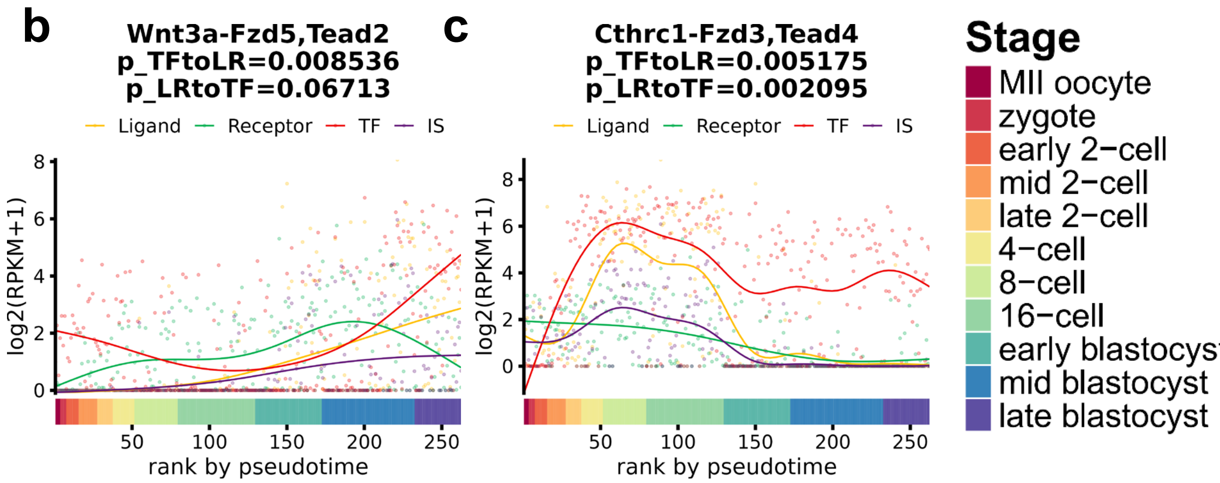

a The list of eLR enriched in the Hippo signaling pathway within clusters 4 and clusters 6. **b** The dynamics of *Wnt3a-Fzd5* and *Tead2.* **c** The dynamics of *Cthrc1-Fzd3* and *Tead4*.

Supplementary Fig. 16: The ratio of the different clusters of eLR regulates tTFs

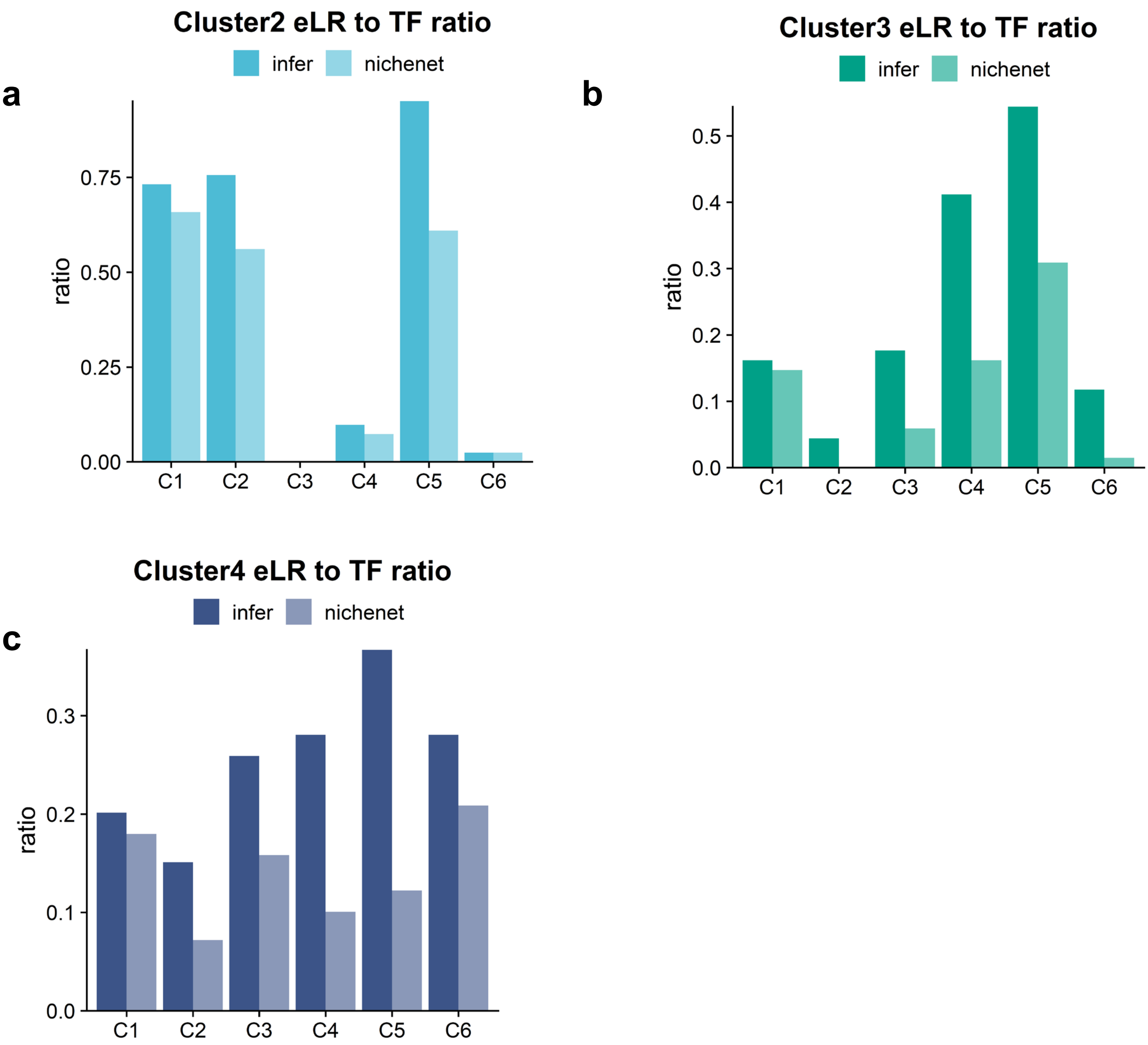

The grouped bar charts depict the proportion of eLRs in each eLR class that can potentially regulate tTFs. The grouped bar chart illustrates the results obtained from TimeTalk (referred to as "infer") and the prior relationships collected in the NicheNet database (referred to as "NicheNet"). These figures are related to Fig. 4c, Fig. 4d, and Fig. 4e.

Supplementary Fig. 17: The percent of the different clusters of tTFs regulate eLR

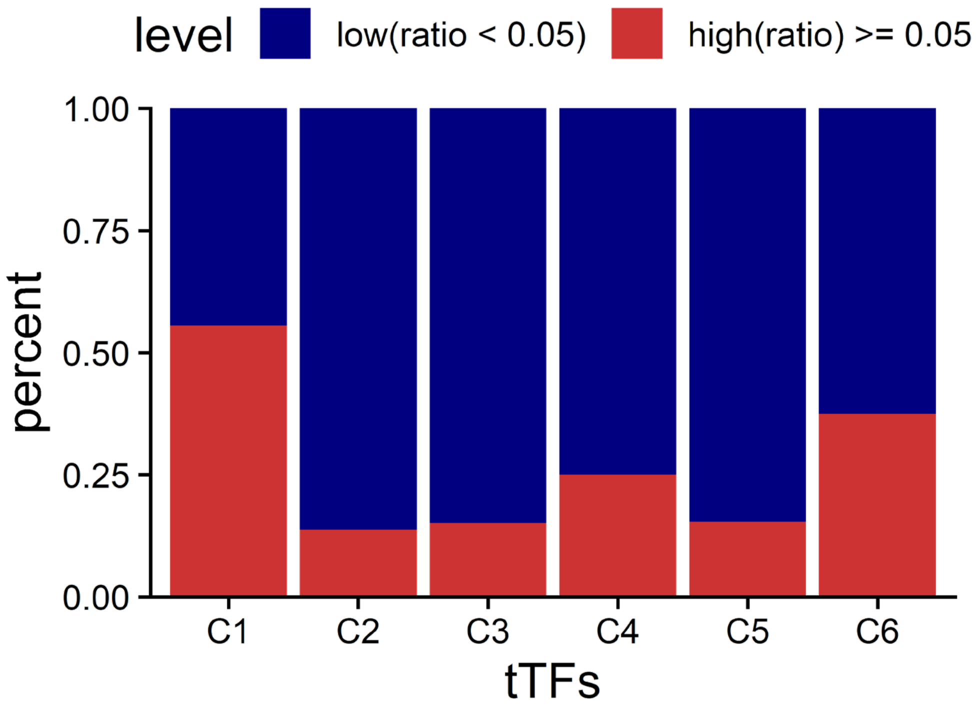

The bar charts demonstrate, in different categories of tTFs, the ratio of tTFs that regulate a large number of eLRs and tTFs that regulate a small number of eLRs. The criterion for a large number is that the proportion of eLR genes in the corresponding target genes of each tTF exceeds 5%, which is considered as tTFs with a large number of eLR targets. This figure is related to Fig. 4f.

Supplementary Fig. 18: The re-analysis of the blastocyst and blastoid scRNA-seq datasets

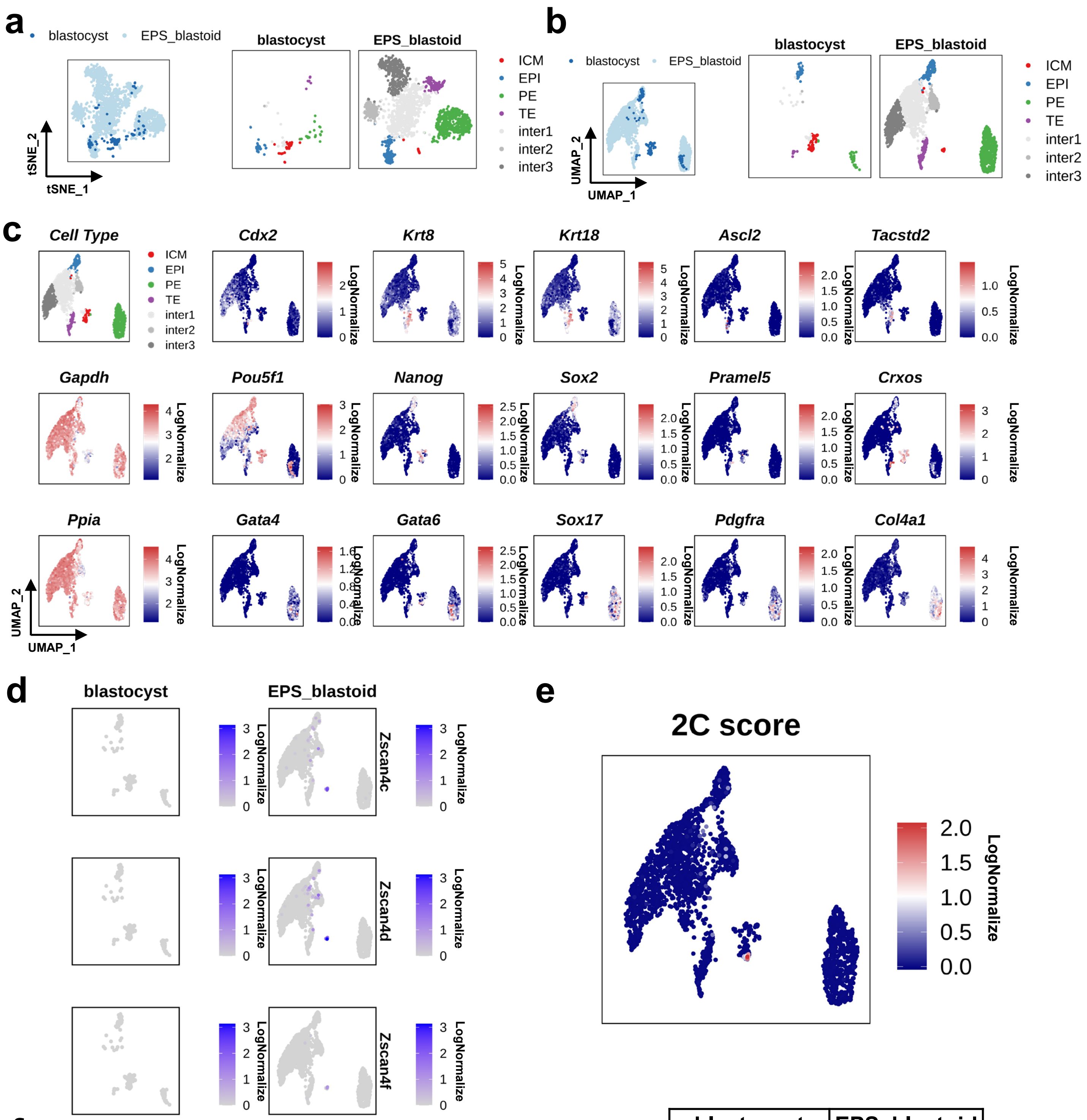

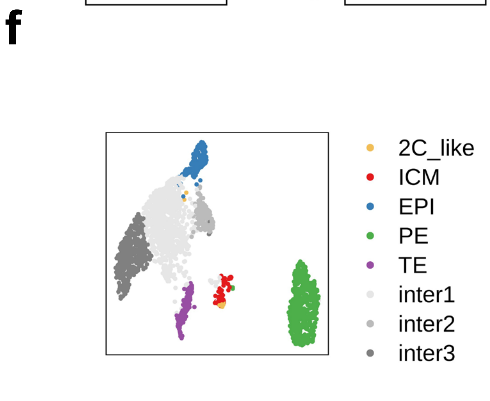

g

	blastocyst	EPS_blastoid
2C_like	0	19
ICM	61	0
EPI	34	140
PE	30	524
TE	9	135
inter1	24	792
inter2	2	166
inter3	0	414

a The t-SNE embedding of the integrated blastocyst and blastoid data. **b** The UMAP embedding of the integrated blastocyst and blastoid data. **c** The feature plot of marker expression of different lineage. **d** The feature plot of 2-cell genes *Zscan4c*, *Zscan4d*, and *Zscan4f*. **e** The feature plot 2C score. The unit for color gradient keys in **c-e** is LogNormalize, which involves dividing the feature counts for each cell by the total counts for that cell and then multiplying it by the scale.factor. This value is then transformed using the natural-log function and adding 1. **f** The cell type annotation of integrated blastoid and blastocyst datasets. **g** The statistics of cell type composition in blastocyst and blastoids.

Supplementary Fig. 19: The sensitivity and reliability analysis of TimeTalk

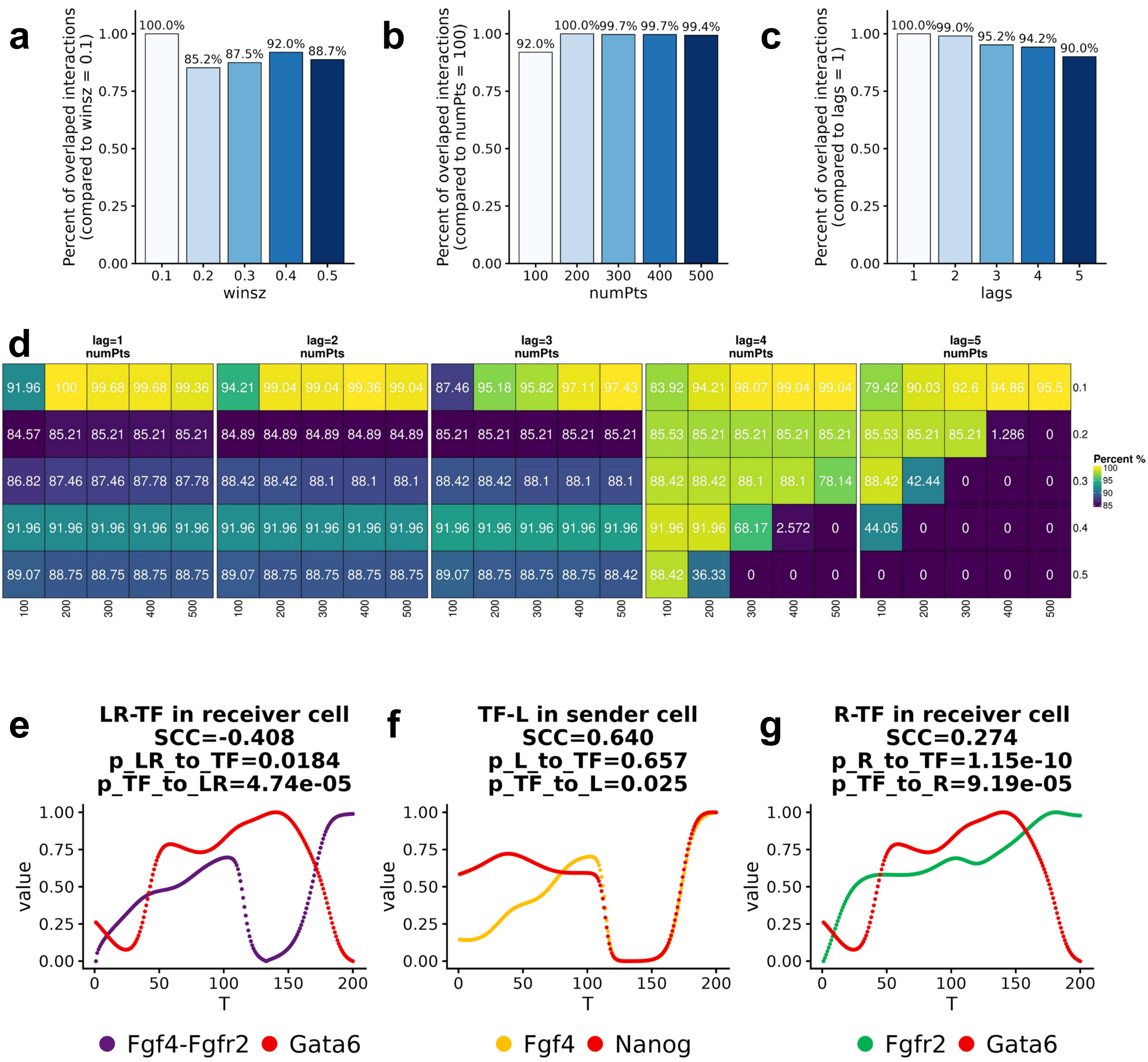

a The barplot displays the overlapped ratio while keeping numPts and lags fixed but varying winsz.**b** The barplot displays the overlapped ratio while keeping winsz and lags fixed but varying lags. **c** The barplot displays the overlapped ratio while keeping winsz and Numpts fixed but varying lags. **d**. The heatmap displays the overlapped ratio for different combinations of winsz, numPts, and lags. **e** The Interpolated dynamic Curves for *Fgf4-Fgfr2* and *Gata6*. **f** The Interpolated dynamic Curves for *Fgf4* and *Nanog.* **g** The interpolated dynamic curves of *Fgfr2* and *Gata6.*

Supplementary Fig. 20: Evaluating the performance of interpolation and Granger causal inference on simulation data without Granger causality

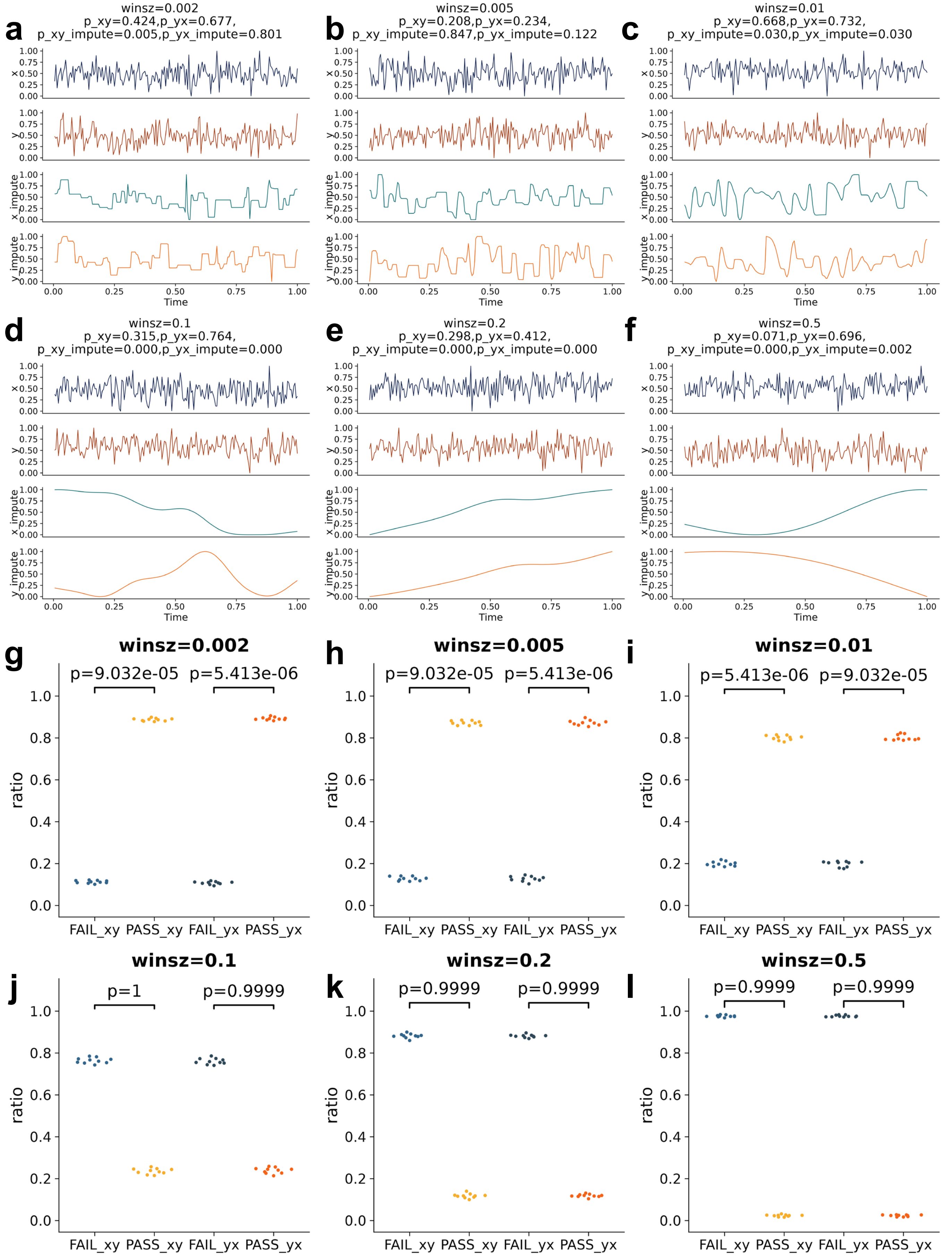

a-f The line plot of time series and interpolated time series, while fixing the other two parameters and varying the winsz. **g-h** The ratio of false positives of 10 rounds simulation results with different winsz. The details are described in Supplementary Note 1.

Supplementary Fig. 21: Evaluating the performance of interpolation and Granger causal inference on simulation data with Granger causality

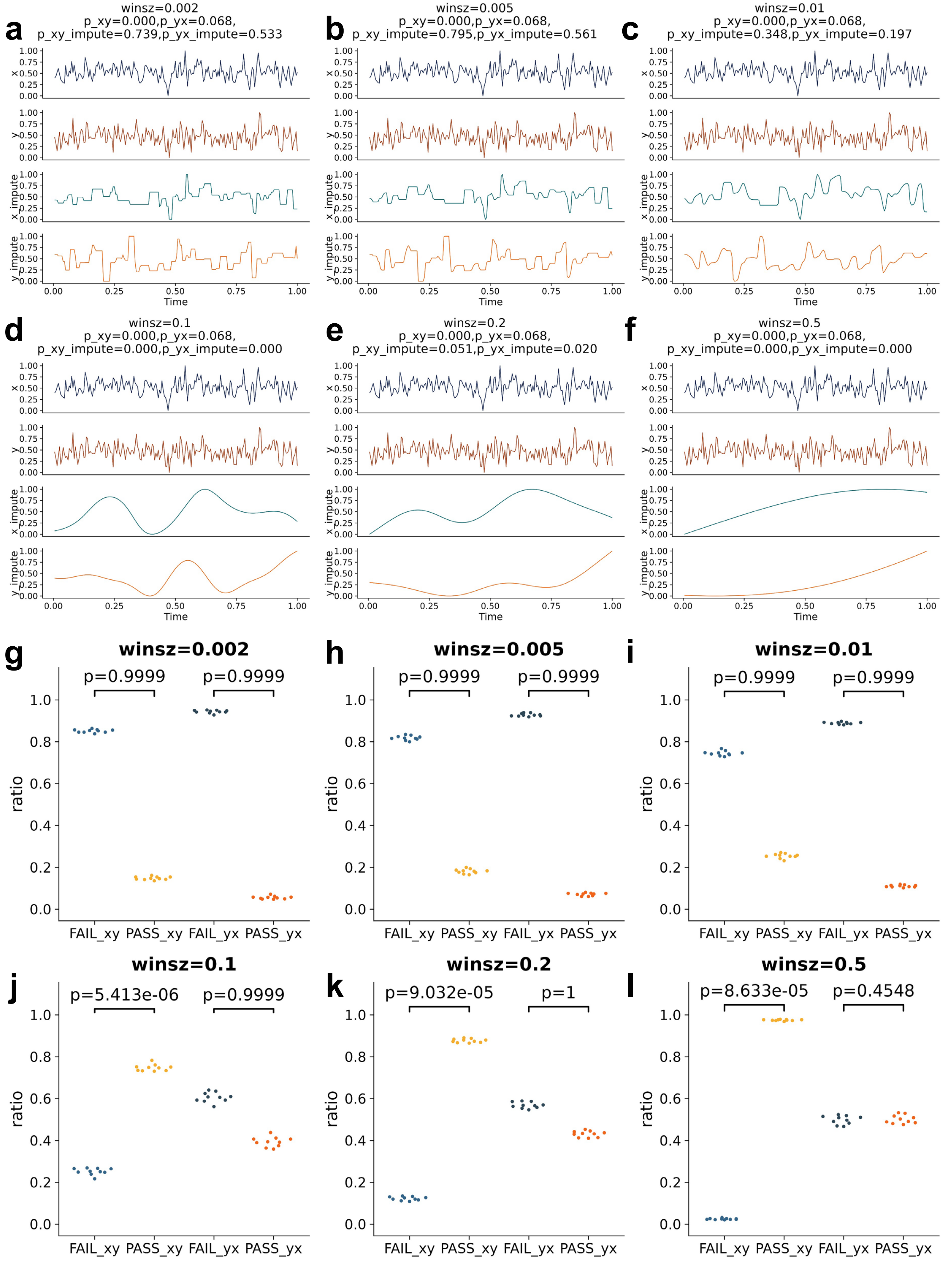

a-f The line plot of time series and interpolated time series, while fixing the other two parameters and varying the winsz. **g-h** The ratio of false positives of 10 rounds simulation results with different winsz. The details are described in Supplementary Note 1.

Supplementary Fig. 22: The co-evolution analysis of LR and non-eLR

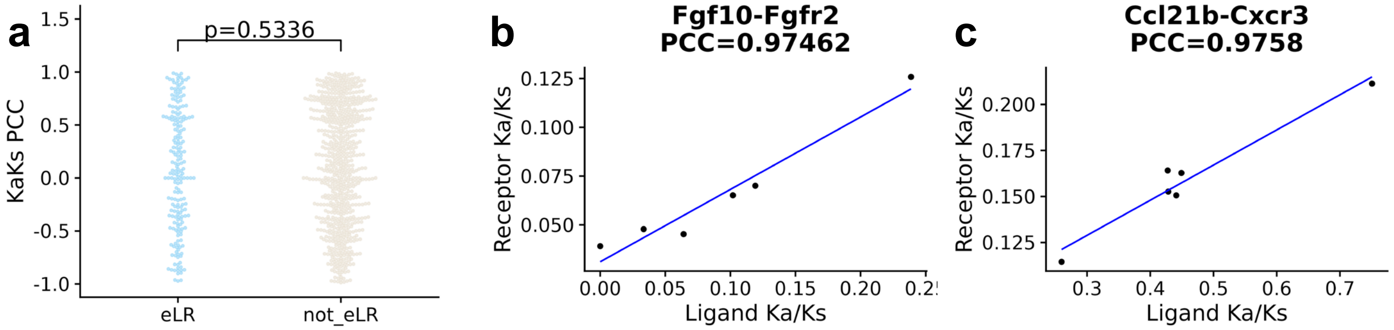

a The boxplot of correlation of the Ka/Ks ratio of ligand gene and receptor gene in the eLR and non-eLR groups. The p-value was calculated by two-sided Wilcoxon test. **b** The correlation of Ka/Ks ratio of eLR *Fgf10-Fgfr2*. **c** The correlation of Ka/KS ratio of non-eLR *Ccl21b-Cxcr3*.
