# Supplementary material for: TimeTalk uses single-cell RNA-seq datasets to decipher cell-cell communication during early embryo development
Source: Commun Biol. 2023 Sep 2;6:901. doi: 10.1038/s42003-023-05283-2 (PMC10475079; doi:10.1038/s42003-023-05283-2)
Supplement: Supplementary file 3 — Description of Additional Supplementary Files [file 42003_2023_5283_MOESM3_ESM.pdf]

Description of Additional Supplementary Files

File Name: Supplementary Data 1
Description: This supplementary dataset provides information on the eLR and tTF characteristics and includes the source data for Fig 2f-g, Fig 3a-d, and Supplementary Fig 13, as referenced in the paper. The README sheet offers detailed explanations of every sheet and column.

File Name: Supplementary Data 2
Description: This supplementary dataset provides information on the relationship between eLR and tTF and includes the source data for Fig 4a-f and Supplementary Fig 16a-c, as referenced in the paper. The README sheet offers detailed explanations of every sheet and column.

File Name: Supplementary Data 3
Description: This supplementary dataset provides information on the various LR-mediated cell-cell communication between EPI and PE which were identified by TimeTalk, and includes the source data for Fig 5c-e. The README sheet offers detailed explanations of every sheet and column.
