# Supplementary material for: TimeTalk uses single-cell RNA-seq datasets to decipher cell-cell communication during early embryo development
Source: Commun Biol. 2023 Sep 2;6:901. doi: 10.1038/s42003-023-05283-2 (PMC10475079; doi:10.1038/s42003-023-05283-2)
Supplement: Supplementary file 7 — Reporting Summary [file 42003_2023_5283_MOESM7_ESM.pdf]

nature portfolio

Corresponding author(s): Prof. Cheng Li: https://orcid.org/0000-0002-1766-1409
Prof. Hebing Chen: https://orcid.org/0000-0003-4102-356X

Last updated by author(s): Jul 30, 2023

Reporting Summary

Nature Portfolio wishes to improve the reproducibility of the work that we publish. This form provides structure for consistency and transparency in reporting. For further information on Nature Portfolio policies, see our Editorial Policies and the Editorial Policy Checklist.

Statistics

For all statistical analyses, confirm that the following items are present in the figure legend, table legend, main text, or Methods section.

n/a	Confirmed	
☐	☒	The exact sample size (n) for each experimental group/condition, given as a discrete number and unit of measurement
☐	☒	A statement on whether measurements were taken from distinct samples or whether the same sample was measured repeatedly
☐	☒	The statistical test(s) used AND whether they are one- or two-sided<br>*Only common tests should be described solely by name; describe more complex techniques in the Methods section.*
☐	☒	A description of all covariates tested
☐	☒	A description of any assumptions or corrections, such as tests of normality and adjustment for multiple comparisons
☐	☒	A full description of the statistical parameters including central tendency (e.g. means) or other basic estimates (e.g. regression coefficient) AND variation (e.g. standard deviation) or associated estimates of uncertainty (e.g. confidence intervals)
☐	☒	For null hypothesis testing, the test statistic (e.g. F, t, r) with confidence intervals, effect sizes, degrees of freedom and P value noted<br>*Give P values as exact values whenever suitable.*
☒	☐	For Bayesian analysis, information on the choice of priors and Markov chain Monte Carlo settings
☒	☐	For hierarchical and complex designs, identification of the appropriate level for tests and full reporting of outcomes
☒	☐	Estimates of effect sizes (e.g. Cohen's d, Pearson's r), indicating how they were calculated

Our web collection on statistics for biologists contains articles on many of the points above.

Software and code

Policy information about availability of computer code

Data collection	https://figshare.com/projects/TimeTalk_CB_manuscript_code_data/174498
Data analysis	https://doi.org/10.6084/m9.figshare.23895780.v6;https://zenodo.org/record/8271645;https://github.com/ChengLiLab/TimeTalk;

For manuscripts utilizing custom algorithms or software that are central to the research but not yet described in published literature, software must be made available to editors and reviewers. We strongly encourage code deposition in a community repository (e.g. GitHub). See the Nature Portfolio guidelines for submitting code & software for further information.

Data

Policy information about availability of data

All manuscripts must include a data availability statement. This statement should provide the following information, where applicable:

- Accession codes, unique identifiers, or web links for publicly available datasets
- A description of any restrictions on data availability
- For clinical datasets or third party data, please ensure that the statement adheres to our policy

The publicly available data on early embryo development used in this paper were obtained from the Gene Expression Omnibus (GEO) database (https://www.ncbi.nlm.nih.gov/geo/). The mouse oocyte scRNA-seq data were obtained from a study by Ramskold et al. with accession number GSE38495. The mouse scRNA-seq data covering the zygote stage to the late blastocyst stage were obtained from a study by Deng et al. with accession number GSE45719. The mouse bulk RNA-seq was obtained from studies by Wu et al. with accession numbers GSE66582. The mouse ZGA inhibition RNA-seq data were obtained from a study by Zhang

et al. with accession number GSE71434. The EPS-generated blastoid scRNA-seq data and natural blastocyst scRNA-seq data were obtained from a study by Li et al. with accession number GSE135701.

Research involving human participants, their data, or biological material

Policy information about studies with human participants or human data. See also policy information about sex, gender (identity/presentation), and sexual orientation and race, ethnicity and racism.

Reporting on sex and gender	n/a
Reporting on race, ethnicity, or other socially relevant groupings	n/a
Population characteristics	n/a
Recruitment	n/a
Ethics oversight	n/a

Note that full information on the approval of the study protocol must also be provided in the manuscript.

Field-specific reporting

Please select the one below that is the best fit for your research. If you are not sure, read the appropriate sections before making your selection.

☒ Life sciences ☐ Behavioural & social sciences ☐ Ecological, evolutionary & environmental sciences

For a reference copy of the document with all sections, see nature.com/documents/nr-reporting-summary-flat.pdf

Life sciences study design

All studies must disclose on these points even when the disclosure is negative.

Sample size	To identify and study eLRs, we collected public early embryo development scRNA-seq datasets from the mouse MII-oocyte stage to the late blastocyst stage accounting 11 stages to ensure that scRNA-seq datasets represented every stage of early embryo development. In addition, In order to study eLRs, we gathered publicly available scRNA-seq datasets of early embryo development in mice. The datasets spanned from the MII-oocyte stage to the late blastocyst stage, covering a total of 11 stages. This ensured that every stage of early embryo development was represented in the datasets. To ensure the data's quality, we also collected public low-input RNA-seq data.
Data exclusions	No data was excluded.
Replication	We provided source code and key data to ensure the replication of our analyes.
Randomization	As this is a public data analysis article, the sample allocation process was not randomized.
Blinding	As this is a public data analysis article, blinding was not available for this research.

Reporting for specific materials, systems and methods

We require information from authors about some types of materials, experimental systems and methods used in many studies. Here, indicate whether each material, system or method listed is relevant to your study. If you are not sure if a list item applies to your research, read the appropriate section before selecting a response.

Materials & experimental systems

n/a	Involved in the study	
☒	☐	Antibodies
☒	☐	Eukaryotic cell lines
☒	☐	Palaeontology and archaeology
☒	☐	Animals and other organisms
☒	☐	Clinical data
☒	☐	Dual use research of concern
☒	☐	Plants

Methods

n/a	Involved in the study	
☒	☐	ChIP-seq
☒	☐	Flow cytometry
☒	☐	MRI-based neuroimaging
